# Real-world evidence of fremanezumab for treating migraine in Japan: a retrospective study

**DOI:** 10.1186/s12883-023-03449-3

**Published:** 2023-11-14

**Authors:** Seiya Ohtani, Narumi Watanabe, Keiko Ihara, Nobuyuki Takahashi, Naoki Miyazaki, Kei Ishizuchi, Ryo Takemura, Satoko Hori, Jin Nakahara, Tsubasa Takizawa

**Affiliations:** 1 Department of Neurology, Keio University School of Medicine, 35 Shinanomachi, Shinjuku-ku, Tokyo, 160-8582 Japan; 2 Division of Drug Informatics, Keio University Faculty of Pharmacy, 1-5-30 Shibakoen, Minato-ku, Tokyo, 105-8512 Japan; 3 Biostatistics Unit, Clinical and Translational Research Center, Keio University Hospital, 35 Shinanomachi, Shinjuku-ku, Tokyo, 160-8582 Japan

**Keywords:** Fremanezumab, Migraine, Real-world evidence

## Abstract

**Background:**

There have been very few real-world studies reported in the literature solely focusing on fremanezumab in Asia. This study aimed to evaluate the efficacy and safety of fremanezumab in a real-world setting in Japan.

**Method:**

This single-centered, observational, retrospective study examined patients with migraines who received four doses of fremanezumab between December 2021 and August 2022 at Keio University Hospital. We assessed the changes in monthly migraine days, responder rates, and migraine-associated symptoms, as well as injection site reactions and adverse events.

**Result:**

Twenty-nine patients were enrolled, wherein 79.3% were women. Compared with those at baseline, the monthly migraine days decreased by 5.9 days at 4 months. The 50% responder rate was 55.2% at 4 months. A total of 57.9%, 47.8%, and 65.0% of patients showed improvement in the severity of photophobia, phonophobia, and nausea/vomiting, respectively. Moreover, injection site reactions were the most common adverse events (55.2%).

**Conclusion:**

Fremanezumab is effective and safe for migraine prevention in Japan. Fremanezumab also improved migraine-associated symptoms in half of the patients.

**Supplementary Information:**

The online version contains supplementary material available at 10.1186/s12883-023-03449-3.

## Background

Migraine is a neurological disorder with a high prevalence (8.4–14.4%) and burden on patients [1–3]. Migraine preventive treatments have improved dramatically with the development of calcitonin gene-related peptide (CGRP)-targeted drugs [4]. The expert consensus statement of the European Headache Foundation guidelines states that monoclonal antibodies (mAbs) targeting the CGRP pathway should be included as first-line treatment options [5]. Clinical studies have indicated the efficacy and safety of fremanezumab, an anti-CGRP mAb (CGRPmAb), in patients with episodic migraine (EM) or chronic migraine (CM) [6–8]. Fremanezumab is the second CGRPmAb to be approved in Japan, along with erenumab (June 2021), after galcanezumab (January 2021). Clinical trials evaluated the efficacy of fremanezumab in Japanese and Korean patients with EM and CM. In an EM study, the 50% responder rate (RR) was 41.3% for monthly dosing and 45.3% for quarterly dosing after 12 weeks. In a CM study, the 50% RR was 29.0% for monthly dosing and 29.1% for quarterly dosing after 12 weeks [9, 10]. A sub-analysis focusing only on Japanese patients has been reported, showing efficacy and safety in Japanese patients [11, 12]. The criteria for administering CGRPmAb differ between Japan and other countries. In Japan, CGRPmAb can be used in patients with ≥ 4 migraine days per month and in those who have undergone treatment with at least one migraine-preventive drug (e.g., lomerizine, propranolol, or valproate), with ineffectiveness, intolerance, or strong concern about side effects [13]. Onabotulinum toxin A, a drug used globally for chronic migraines, has not been approved in Japan. Comparing clinical trials with real-world (RW) studies, clinical trials more likely to have a relatively homogeneous population, and the data quality is higher because headaches are assessed in detail using electronic headache diaries. Many trials excluded patients with special medical conditions or a high number of prophylactic drug failures (some trials included a large number of prophylactic drug failures) [6–10]. In many cases, no other prophylaxis is used during clinical trials; if used, it is used only in a small number of cases. By contrast, patients in RW studies have diverse backgrounds. In RW studies, the effect of CGRPmAb is often greater than that in clinical trials, partly because no placebo is used for comparison. Thus, it is important to construct RW evidence that reflects daily practices regarding CGRPmAb.

Two RW studies reported the efficacy and safety of fremanezumab in Italy [14, 15]. The 50% RR at 3 months was 64.2% [14]. We have recently published RW evidence paper on galcanezumab from Japan, which showed a 50% responder rate of 61.5% at 3 months [16]. There have been RW evidence studies on CGRPmAbs, including fremanezumab, from Japan [17, 18]. To the best of our knowledge, only one other RW study solely focusing on fremanezumab have been published in international journal from Japan or Asia [19]. Differences in race or criteria for the use of fremanezumab may cause dissimilar results between Japanese studies and those from other countries. Therefore, this study aimed to determine the efficacy and safety of fremanezumab in RW settings in Japan.

## Methods

### Study design

We conducted a single-center, observational, retrospective cohort study. This study was approved by the Ethics Committee of the Keio University School of Medicine (approval number:20211144), Tokyo, Japan. The patients were informed about this observational study via the institute’s website and could opt out of the study. The need for informed consent was waived by the Ethics Committee of the Keio University School of Medicine in accordance with national regulations (Ethical Guidelines for Medical and Biological Research Involving Human Subjects) [16]. The patients included in this study partly overlapped with previously reported responder analysis study that gathered information about fremanezumab, galcanezumab, and erenumab [20].

### Patients

Patients were administered fremanezumab 225 mg subcutaneously (i.e., a single dose) at the first administration in our hospital. Next, the patients were administered 225 mg of subcutaneous fremanezumab monthly or 675 mg quarterly at the second visit, according to their preference. We started with a single dose of fremanezumab instead of three because each dose costs approximately 12,350 yen (88 USD as of June 2023) for most patients subscribing to the Japanese insurance system. During the study period, fremanezumab was only permitted in the syringe and not by self-injection in Japan; therefore, patients who did not prefer monthly visits to the hospital tended to choose quarterly dosing from the second dose.

**Fig. 1:**
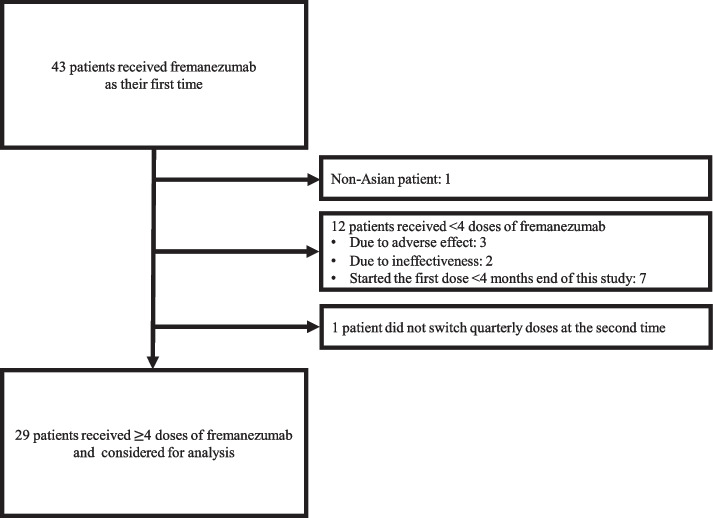
Patient selection

The inclusion criteria were as follows: treatment with fremanezumab for 4 months as the first CGRPmAb (de novo) either via monthly injections of fremanezumab 225 mg for four times or a dose of fremanezumab 225 mg at the first administration and a quarterly dose of fremanezumab 675 mg at the second administration from the headache group of the Keio University Hospital between December 2021 (when the drug became available at the hospital) and August 2022; fulfillment of the diagnostic criteria for migraine, including probable migraine, according to the International Classification of Headache Disorders, 3rd edition (ICHD-3); and age ≥ 18 years. The patients were diagnosed with migraine by a headache specialist. Non-Asian patients were excluded (Fig. 1).

### Research items

We retrospectively collected demographic data, medical history, family history of headache, and migraine characteristics. Generalized Anxiety Disorder-7 (GAD-7) [21, 22] and Patient Health Questionnaire-9 (PHQ-9) [23] were assessed before administering CGRPmAbs to determine the extent of anxiety and depression, respectively. We also collected migraine-preventive drug data, including the drugs administered (lomerizine, propranolol, valproate, amitriptyline, or topiramate), the use or non-use of preventive drugs at the first dose, and the handling of preventive drugs at the first dose [16].

Headache specialists explained the criteria for migraine based on the ICHD-3 to all patients, who were asked to track their headache and migraine days, including probable migraine days. Patients completed a questionnaire on monthly migraine days (MMD), monthly headache days (MHD), monthly days with acute medication use (AMD), pain intensity (0–10 numerical rating scale (NRS)), and associated symptoms (none, mild, moderate or severe) at baseline and after the first, second, third, and fourth months (Supplementary Fig. 1). The patients who injected quarterly doses recorded questionnaire monthly as well. Headache specialists verified the accuracy and reliability of the completed questionnaires by interviewing and occasionally reviewing each patient’s headache diary [16].

Information on the injection sites, reactions (pain, redness, swelling, numbness, or others), severity, and other adverse reactions were also collected in the questionnaire. The patients were asked about their satisfaction levels at 4 months after receiving fremanezumab [16].

### Outcomes

We investigated the efficacy of the therapy by measuring the changes in MMD, MHD, AMD, NRS scores, and associated symptoms. The primary endpoints were a change in the MMD from baseline and 50% responder rate (RR) based on MMD. The secondary endpoints were changes from baseline in MHD; AMD; NRS; 25%, 75%, and 100% RR; and associated symptoms. We defined an improvement in associated symptoms as a reduction in symptom severity (e.g., severe to mild), and a disappearance as a disappearance of symptoms (e.g., severe to none). For safety, we investigated the injection sites and reactions, and other adverse events [16].

### Statistical analysis

Data are presented as number (percent) and mean ± standard deviation. Differences from baseline in MMD, MHD, AMD, and NRS and their least-squares means were analyzed using the mixed-effects model for repeated measures, with time as a fixed effect and individual as a random effect. The correlation structure was defined as unstructured. Normality was assessed visually using residual plots. We did not impute missing data. The statistical analyses were performed using SAS version 9.4 (SAS Institute Inc., Cary, NC, USA). Statistical significance was set at *p* < 0.05 [16].

## Results

### Patients

Forty-three patients who experienced migraine received fremanezumab for the first time between December 2021 and August 2022. We excluded one non-Asian patient. Twelve patients received fremanezumab for < 4 months: 5 discontinued fremanezumab due to adverse effects (constipation, pruritus, and skin rash) or ineffectiveness, and 7 started fremanezumab later than 4 months before the end of the study period. We excluded one patient who switched from monthly to quarterly doses during the third administration. Twenty-nine patients were considered eligible for the efficacy and safety analyses (225 mg monthly only, *n* = 19; 225 mg/625 mg quarterly, *n* = 10) (Fig. 1).

### Baseline characteristics

Most of the patients were women, and the mean age was 47.2 ± 12.4 (26–72) years. The mean MMD, MHD, and AMD were 12.6 ± 7.2 days/month, 14.7 ± 7.1 days/month, and 10.5 ± 7.3 days/month, respectively. At baseline, 34.5% and 24.1% of patients were diagnosed with CM and MOH, respectively (Table 1).

**Table 1:** Demographic and clinical characteristics of patients

Characteristics	EM (*n* = 19)	CM (*n* = 10)	All (*n* = 29)
Age, years	50.7 ± 11.2	40.5 ± 8.6	47.2 ± 12.4
Sex, female	15 (78.9)	8 (80.0)	23 (79.3)
BMI, kg/m2	21.4 ± 4.0	23.6 ± 7.2	22.2 ± 5.3
Onset age, years	24.4 ± 11.2	15.7 ± 9.4	21.4 ± 11.3
Disease history, years	26.3 ± 14.2	24.8 ± 13.3	25.8 ± 13.7
NRS	5.6 ± 1.6	5.9 ± 1.1	5.7 ± 1.4
Migraine characteristics			
Unilateral pain	15 (78.9)	6 (60.0)	21 (72.4)
Pulsating pain	11 (57.9)	6 (60.0)	17 (58.6)
Aggravation by routine physical activity	15 (78.9)	10 (100.0)	25 (86.2)
MMD	8.8 ± 2.8	19.7 ± 7.6	12.6 ± 7.2
MHD	10.5 ± 2.5	22.7 ± 5.9	14.7 ± 7.1
AMD	7.0 ± 3.9	17.0 ± 7.8	10.5 ± 7.3
Medication-overuse headache	0 (0.0)	7 (70.0)	7 (24.1)
Aura	3 (15.8)	1 (10.0)	4 (13.8)
Associated symptoms			
Photophobia	12 (63.2)	7 (70.0)	19 (65.5)
Phonophobia	16 (84.2)	7 (70.0)	23 (79.3)
Nausea/vomiting	13 (68.4)	7 (70.0)	20 (69.0)
Psychiatric past history	5 (26.3)	5 (50.0)	10 (34.5)
GAD-7 ≥ 5	7 (36.8)	6 (60.0)	13 (44.8)
GAD-7 ≥ 10	1 (5.3)	3 (30.0)	4 (13.8)
PHQ-9 ≥ 5	9 (47.4)	8 (80.0)	17 (58.6)
PHQ-9 ≥ 10	2 (10.5)	3 (30.0)	5 (17.2)
Family history of headache	12 (63.2)	7 (70.0)	19 (65.5)

Data are presented as n (%) or mean ± standard deviation

*EM* episodic migraine, *CM* chronic migraine, *BMI* body mass index, *NRS* numerical rating scale, *MMD* monthly migraine day, *MHD* monthly headache day, *AMD* monthly acute medication days, *GAD-7* General Anxiety Disorder-7, *PHQ-9* 9-item Patient Health Questionnaire

### Preventive drugs

In terms of previous use of other migraine preventives, 22 (75.9%), 3 (10.3%), 18 (62.1%), 7 (24.1%), and 4 (13.8%) patients had used lomerizine, propranolol, valproate, amitriptyline, and topiramate, respectively. Eleven (37.9%) patients used only one preventive drug, and the mean number of previous migraine preventives used was 1.9 ± 0.8. Twenty (69.0%) patients were using migraine prophylaxis at the time of initiating fremanezumab. Approximately half of the patients discontinued migraine prevention after the first dose of fremanezumab (Table 2).

**Table 2:** Preventive drugs used in studied patients

	EM (*n* = 19)	CM (*n* = 10)	All (*n* = 29)
Types of preventive drugs			
Lomerizine	14 (73.7)	8 (80.0)	22 (75.9)
Propranolol	2 (10.5)	1 (10.0)	3 (10.3)
Valproate	11 (57.9)	7 (70.0)	18 (62.1)
Amitriptyline	5 (26.3)	2 (20.0)	7 (24.1)
Topiramate	2 (10.5)	2 (20.0)	4 (13.8)
Number of preventive drug(s) used			
1	9 (47.4)	2 (20.0)	11 (37.9)
2	6 (31.6)	6 (60.0)	12 (41.4)
3	3 (15.8)	2 (20.0)	5 (17.2)
4	1 (5.3)	0 (0.0)	1 (3.4)
5	0 (0.0)	0 (0.0)	0 (0.0)
Mean	1.8 ± 0.9	2.0 ± 0.7	1.9 ± 0.8
Use of preventive drug at the first dosage			
No	6 (31.6)	3 (30.0)	9 (31.0)
Yes	13 (68.4)	7 (70.0)	20 (69.0)
Discontinued	5 (26.3)	4 (40.0)	9 (31.0)
Continued	8 (42.1)	3 (30.0)	11 (37.9)

Data are presented as n (%) or mean ± standard deviation

The preventive drugs assessed are lomerizine, propranolol, valproate, amitriptyline, and topiramate

*EM* episodic migraine, *CM* chronic migraine

### Efficacy of fremanezumab for headache

#### All

**Fig. 2:**
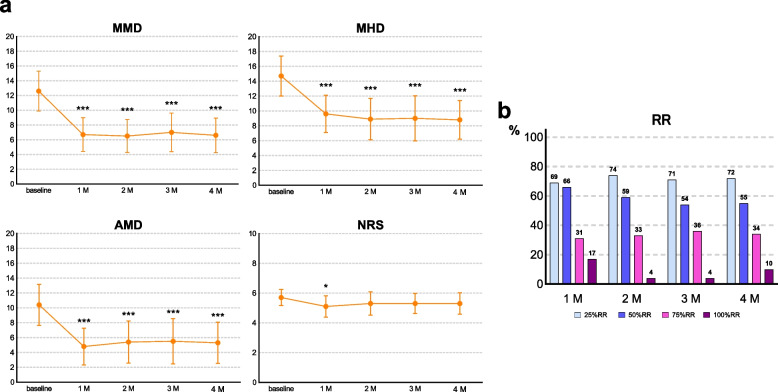
Efficacy of fremanezumab in all patients. **a** Changes in MMD, MHD, AMD, and NRS from baseline. *Significant difference compared with baseline. * adjusted *p* < 0.05; **adjusted *p* < 0.01; ***adjusted *p* < 0.001. Error bars represent 95% confidence interval. Abbreviations: MMD, monthly migraine days; MHD, monthly headache days; AMD, monthly days with acute medication use; NRS, numerical rating scale; 1 M, 1 month; 2 M; 2 months; 3 M; 3 months; 4 M, 4 months. **b** Responder rates. Proportion of patients with responder rates of 25%, 50%, 75%, and 100%. Abbreviations: 1 M, 1 month; 2 M, 2 months; 3 M, 3 months; 4 M, 4 months; RR, responder rate

At baseline, the average MMD was 12.6 ± 7.2 days/month. Compared with this baseline, MMD decreased by 5.9 days (95% confidence interval [CI], 3.6–8.2; *p* < 0.001) at 1 month, 6.0 days (95% CI, 3.8–8.3; *p* < 0.001) at 2 months, 5.5 days (95% CI, 3.1–7.9; *p* < 0.001) at 3 months, and 5.9 days (95% CI, 3.1–8.7; *p* < 0.001) at 4 months. The MHD, AMD, and NRS scores were significantly reduced at 1 month compared to those at baseline (Fig. 2a). The 50% RR was 65.5% (95%CI, 45.7–82.1) at 1 month, 59.3% (95%CI, 38.8–77.6) at 2 months, 53.6% (95%CI, 33.9–72.5) at 3 months, and 55.2% (95%CI, 35.7–73.6) at 4 months; the 100% RR was 10.3% (95%CI, 2.2–27.4) at 4 months (Fig. 2b).

#### EM (*n* = 19)

**Fig. 3:**
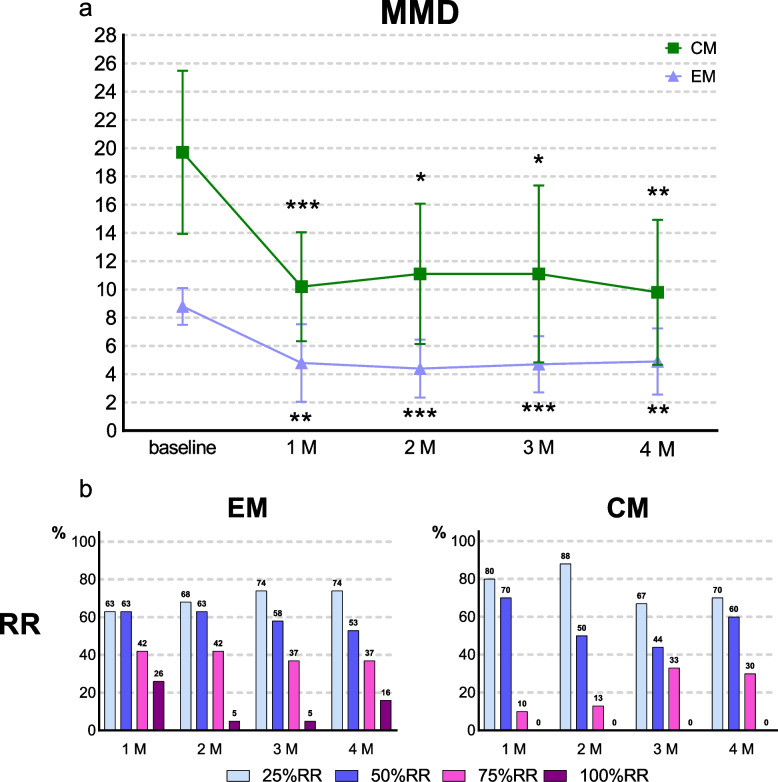
Efficacy of fremanezumab in patients with EM and CM. **a** Changes in MMD from baseline. *Significant difference compared with baseline. * adjusted *p* < 0.05; **adjusted *p* < 0.01; ***adjusted *p* < 0.001. Error bars represent 95% confidence interval. Abbreviations: MMD, monthly migraine day; EM, episodic migraine; CM, chronic migraine; 1 M, 1 month; 2 M, 2 months; 3 M, 3 months; 4 M, 4 months. **b** Responder rates. Proportion of patients with responder rates of 25%, 50%, 75%, and 100%. Abbreviations: EM, episodic migraine; CM, chronic migraine; 1 M, 1 month; 2 M, 2 months; 3 M, 3 months; 4 M, 4 months; RR, responder rate

At baseline, the mean MMD was 8.8 ± 2.8 days/month. Compared with this baseline, MMD decreased by 4.0 days (95% CI, 1.5–6.5; *p* = 0.003) at 1 month, 4.4 days (95% CI, 2.4–6.4; *p* < 0.001) at 2 months, 4.1 days (95% CI, 2.2–6.0; *p* < 0.001) at 3 months, and 3.8 days (95% CI, 1.3–6.3; *p* = 0.005) at 4 months (Fig. 3a). The 50% RR was 63.2% (95% CI, 38.4–83.7) at 1 month, 63.2% (95% CI, 38.4–83.7) at 2 months, 57.9% (95% CI, 33.5–79.7) at 3 months, and 52.6% (95% CI, 28.9–75.6) at 4 months; the 100% RR was 15.8% (95% CI, 3.4–39.6) at 4 months (Fig. 3b).

#### CM (*n* = 10)

At baseline, the average MMD was 19.7 ± 7.6 days/month. Compared with this baseline, MMD decreased by 9.5 days (95% CI, 5.2–13.8; *p* < 0.001) at 1 month, 8.6 days (95% CI, 0.8–16.5; *p* = 0.036) at 2 months, 8.6 days (95% CI, 1.1–16.0; *p* = 0.030) at 3 months, and 9.9 days (95% CI, 3.2–16.6; *p* = 0.009) at 4 months (Fig. 3a). The 50% RR was 70% (95% CI, 34.8–93.3) at 1 month, 50.0% (95% CI, 15.7–84.3) at 2 months, 44.4% (95% CI, 13.7–78.8) at 3 months, and 60.0% (95% CI, 26.2–87.8) at 4 months; the 100% RR was not observed (Fig. 3b).

#### Quarterly (*n* = 10)

**Fig. 4:**
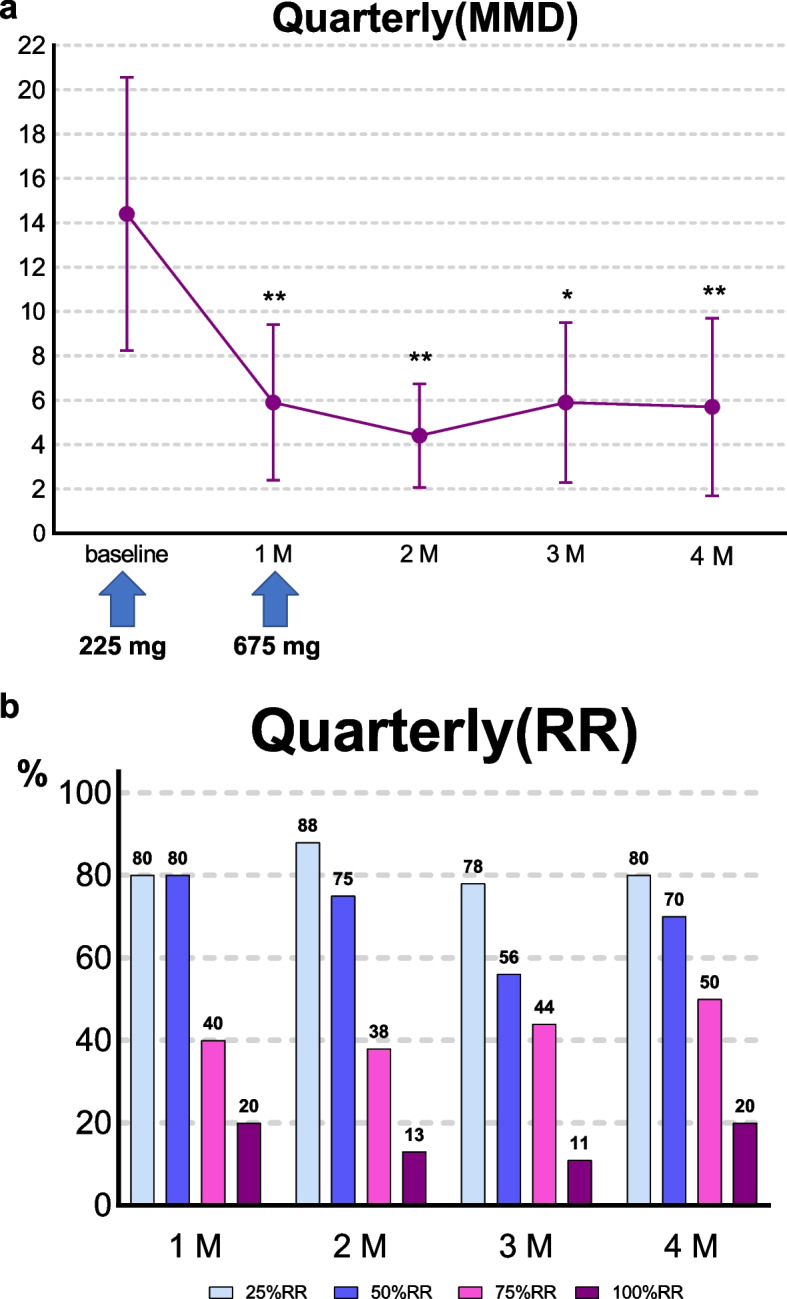
Efficacy of fremanezumab for quarterly patients. Patients received fremanezumab (225 mg) as the first dose and a quarterly dose of fremanezumab (675 mg) as the second dose. **a** Changes in MMD from baseline. *Significant difference compared with baseline. * adjusted *p* < 0.05; **adjusted *p* < 0.01; ***adjusted *p* < 0.001. Error bars represent 95% confidence interval. Abbreviations. MMD, monthly migraine days; 1 M, 1 month; 2 M, 2 months; 3 M, 3 months; 4 M, 4 months. **b** Responder rates. Proportion of patients with responder rates of 25, 50, 75, and 100

5 EM patients (26.3%) and 5 CM patients (50.0%), comprising 34.5% of studied patients, chose one dose of fremanezumab as the first dose and a quarterly dose of fremanezumab as the second dose. At baseline, the average MMD was 14.4 ± 8.6 days/month. Compared with this baseline, MMD decreased by 8.5 days (95% CI, 4.0–13.0; *p* = 0.002) at 1 month, 9.7 days (95% CI, 4.3–15.1; *p* = 0.003) at 2 months, 8.4 days (95% CI, 2.2–14.6; *p* = 0.015) at 3 months, and 8.5 days (95% CI, 3.6–13.4; *p* = 0.004) at 4 months (Fig. 4a). The 50% RR was 80.0% (95% CI, 44.4–97.5) at 1 month, 75.0% (95% CI, 34.9–96.8) at 2 months, 55.6% (95% CI, 21.2–86.3) at 3 months, and 70.0% (95% CI, 34.8–93.3) at 4 months; the 100% RR was 20.0% (95% CI, 2.5–55.6) at 4 months (Fig. 4b). At 1 month, the 50% RR of the patients who received one dose a month and the following quarterly doses was higher than that of those who received only monthly doses, but the result was not statistically significant (80% (95% CI, 44.4–97.5) vs 57.9% (95% CI, 33.5–79.7); *p* = 0.234) (Supplementary Fig. 2).

### Associated symptoms

**Fig. 5:**
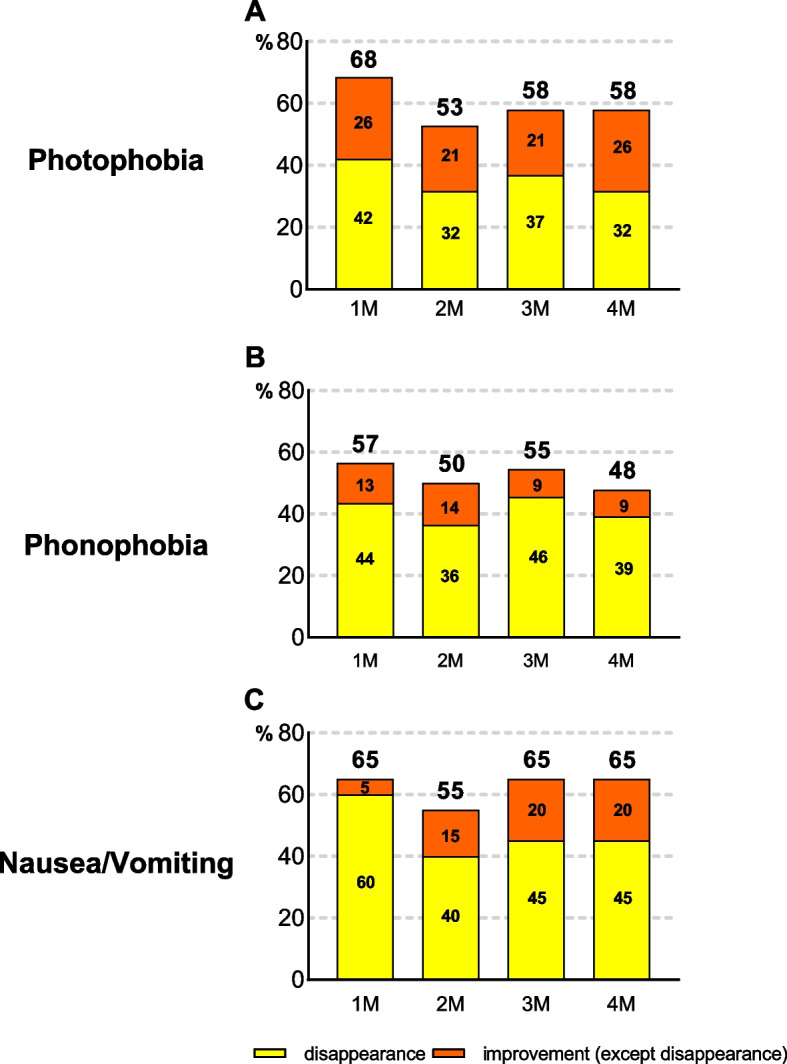
Improvement and disappearance of the associated symptoms. **A** Photophobia, **B** Phonophobia, and **C** Nausea/vomiting

An improvement in photophobia was recorded in 68.4% (95% CI, 43.4–87.4), 52.6% (95% CI, 28.9–75.6), 57.9% (95% CI, 33.5–79.7), and 57.9% (95%CI, 33.5–79.7) of the patients at 1, 2, 3, and 4 months, respectively. An improvement in phonophobia was recorded in 56.5% (95% CI, 34.5–76.8), 50.0% (95% CI, 28.2–71.8), 54.5% (95% CI, 32.2–75.6), and 47.8% (95% CI, 26.8–69.4) of the patients at 1, 2, 3, and 4 months, respectively. An improvement in nausea/vomiting was recorded in 65.0% (95% CI, 40.8–84.6), 55.0% (95% CI, 31.5–76.9), 65.0% (95%CI, 40.8–84.6), and 65.0% (95% CI, 40.8–84.6) at 1, 2, 3, and 4 months, respectively (Fig. 5). Photophobia, phonophobia, and nausea/vomiting disappeared in 31.6% (95% CI, 12.6–56.6), 39.1% (95% CI, 19.7–61.5), and 45.0% (95% CI, 23.1–68.5) of the patients with symptoms at baseline, respectively, at 4 months (Fig. 5).

### Safety

All patients received fremanezumab at least once on the forearm (Table 3a). Eight (28.6%), eight (28.6%), six (31.6%) and, six (31.6%) patients showed injection site reactions after the first, second, third, and fourth injections, respectively. During 4 months, 16 (55.2%) patients had at least one episode of injection site reaction. The injection site reactions were mild to moderate, except in two patients who experienced severe redness and swelling (Table 3b). Swelling was the most commonly reported injection site reaction (Table 3c). Adverse events other than injection site reactions are shown in Table 4.

**Table 3:** Injection site and injection site reaction after fremenezumab

(a) Injection site						
Dose	Abdomen	Forearm	n			
First	0	29 (100.0)	29			
Second	6 (21.4)	27 (96.4)	28			
Third	0	19 (100.0)	19			
Forth	0	19 (100.0)	19			
(b) Degree of Injection site reaction						
Dose	None	Mild	Moderate	Severe	n	
First	20 (71.4)	7 (25.0)	1 (3.6)	0	28	
Second	20 (71.4)	6 (21.4)	1 (3.6)	1 (3.6)	28	
Third	13 (68.4)	3 (15.8)	2 (10.5)	1 (5.3)	19	
Forth	13 (68.4)	3 (15.8)	3 (15.8)	0	19	
(c) Types of Injection site reaction						
Dose	Pain	Redness	Swelling	Numbness	Others	n
First	3 (10.7)	1 (3.6)	6 (21.4)	2 (7.1)	0	28
Second	3 (10.7)	3 (10.7)	6 (21.4)	0	1 (3.6)	28
Third	2 (10.5)	4 (21.1)	4 (21.1)	0	2 (10.5)	19
Forth	1 (5.3)	4 (21.1)	6 (31.6)	0	3 (15.8)	19

Data are presented as n (%)

**Table 4:** Other adverse events

Other adverse events	n(%)
Back pain	1 (3.4)
Constipation	1 (3.4)
Headache	1 (3.4)
Lightheadeness	1 (3.4)
Nausea	1 (3.4)
Palpitation	1 (3.4)
Scalp pain	1 (3.4)

Data are presented as n (%)

### Satisfaction level

Among patients who were administered fremanezumab, 15 (51.7%) were very satisfied with the therapy, 10 (34.5%) were somewhat satisfied, and 4 (13.8%) were unsatisfied.

## Discussion

To the best of our knowledge, this is the second RW study focusing solely on fremanezumab in Asian patients with migraine to be reported in an international journal [19]. Our results suggest that fremanezumab is effective and safe in the Japanese population.

The efficacy of fremanezumab has been confirmed in randomized controlled trials, such as the HALO-EM and CM studies. In the HALO-EM study, the change in MMD was -3.7 days/month for monthly dosing and -3.4 days/month for quarterly dosing, and the 50% RR was 47.7% for monthly dosing and 44.4% for quarterly dosing [6]. In the HALO-CM study, the change in MHD was -4.6 days/month for monthly dosing and -4.3 days/month for quarterly dosing, and the 50% RR was 40.8% for monthly dosing and 37.6% for quarterly dosing [8]. clinical trials evaluated the efficacy of fremanezumab in Japanese and Korean patients with EM and CM. In the EM study, the change in MMD was -4.0 days/month for monthly dosing and -4.0 days/month for quarterly dosing, and the 50% RR was 41.3% for monthly dosing and 45.3% for quarterly dosing [10]. In the CM study, the change in headache days of at least moderate severity per month was -4.1 days/month for monthly dosing and -4.1 days/month for quarterly dosing, and the 50% RR was 29.0% for monthly dosing and 29.1% for quarterly dosing [9]. The efficacy (EM: -3.8 MMD and 50% RR of 52.6%; CM: -9.9 MMD and 50% RR of 60.0%) was better in this RW study than that in the above clinical trials.

There was a multicenter RW cohort study on fremanezumab efficacy published in Italy [14], wherein 67.9% of the patients had CM, whereas only 34.5% of the patients in the present study had CM. At baseline, MMD/MHD was 17.0 ± 6.2 in the Italy study, while MMD was 12.6 ± 7.2 in this study. In terms of the unsuccessful previous preventive drugs, the percentage of patients with $\ge$ 3 failures was 92.0% in Italy and 20.6% in this study. Onabotulinumtoxin A was used in previous studies (Italy, 6.9%) but not in the current study. As for the 50% RR at 3 months, the 50% RR was considered as 64.2% in the Italian study [14]. The 50% RR at 3 months was 53.6% in the present study. Previous studies reported that the total number of prior treatment failures is a negative predictor of response [18, 20, 24, 25]. Therefore, the 50% RR in this study would have been expected to be higher than that reported by the Italian study, but the numbers were similar between two studies. This may be due to the difference in the way of assessing endpoints (i.e. MMD/MHD for EM/CM in Italian study, and MMD in our study).

There have been RW studies on CGRPmAbs, including fremanezumab, in Japan [17, 18]. In Suzuki's study (228 patients: 45 erenumab, 123 fremanezumab, and 60 galcanezumab), the 50% RR at 3 months was 48.2% [18], which is similar to our study (53.6%). Recently, another paper from Japan solely focusing on fremanezumab has been published [19]. As for the 50% RR at 4 months, our study and the recent study showed similar numbers (56.6% and 55.2%) suggesting adequate efficacy of fremanezumab in Japanese real-world population [19].

In our study, 10 patients started with fremanezumab 225 mg as the first dose and changed to fremanezumab 675 mg (quarterly dosing) from the second month. These patients showed sustained benefit with a high 50% RR (1 month: -8.5 MMD and 50% RR of 80.0%; 4 months: -8.5 MMD and 50% RR of 70.0%). In this RW study, it is not recommended to compare monthly vs quarterly dosing due to the possible selection bias. The dosing change was based on patients’ preferences. Those who preferred a quarterly dose in the beginning may have chosen to continue with the monthly dose if they were suspicious of the effect at 1 month, ending up in the monthly injection group.

All three associated symptoms improved in the present study. In the Phase 3 HALO-EM study, fremanezumab reduced all three associated symptoms after 4 weeks. Fremanezumab significantly reduced the monthly average number of days with photophobia and phonophobia and nausea or vomiting from baseline for monthly (-3.0 ± 0.23 days, -3.0 ± 0.22 days, -2.1 ± 0.19 days) and quarterly (-2.8 ± 0.23 days, -2.7 ± 0.22 days, -1.9 ± 0.19 days) dosing strategies during the 12-week treatment period [26]. Efficacy for associated symptoms was also observed at 1 month in this study. The rates of improvement in the associated photophobia, phonophobia, and nausea/vomiting were 68.4%, 56.5%, and 65.0% at 1 month, respectively, and 57.9%, 47.8%, and 65.0% at 4 months, respectively.

As for adverse reactions, injection site reactions were reported more frequently (up to 55.2% reported at least once) in our study compared to other real-world studies (< 9%) [14, 15, 19]. This may be due to the difference in study design. We asked about the status of adverse events especially about injection site reaction using questionnaire for each visit. We speculate that the use of a questionnaire probably increased the reporting rate of adverse event.

No serious adverse events were observed in patients who received fremanezumab, and the most frequent adverse events were injection site reactions. However, three patients discontinued fremanezumab due to side effects (constipation, pruritus, and skin rash). In our previous galcanezumab RW study, the most frequent injection reaction was pain, while in this study, the most frequent injection reaction was swelling [16]. This difference may be due to differences in the injection devices (galcanezumab, auto-injector; fremanezumab, syringe at the time of the study).

Satisfaction rate (very satisfied and somewhat satisfied) was high at 86.2%. High satisfaction rates with anti-CGRPmAbs have also been reported [27]. Considering those who discontinued fremanezumab owing to adverse events and ineffectiveness, the satisfaction rate was still high at 73.5% in our current study. These numbers are compatible with our report on satisfaction levels with galcanezumab (74.5%) [16].

This study has several strengths. It is the second RW study from Asia that describes the efficacy and safety of fremanezumab solely in migraines. Moreover, to our knowledge, this is the first study to analyze the efficacy of changing the dosage from monthly to quarterly. Additionally, we analyzed the improvement in migraine-associated symptoms with fremanezumab, which has not been previously studied in the RW setting.

However, this study had limitations including a small sample size, retrospective nature, single-center design, short observation period of 4 months, and selection bias regarding different schedules of administration. The primary endpoint (migraine days) was mainly assessed with questionnaires and not by the actual headache diaries, which were only checked in some cases [16, 20]. Thus, further studies are necessary to elucidate the effects of fremanezumab.

## Conclusions

This study revealed that fremanezumab is effective and safe for migraine prevention in Japan. Fremanezumab also improved migraine-associated symptoms in approximately half of the patients.

### Supplementary Information

**Additional file 1: Supplementary Figure 1.** Questionnaire.

**Additional file 2: Supplementary Figure 2.** Responder rates at 1M for monthly and quarterly patients.

## Data Availability

The datasets analyzed during the current study are available from the corresponding author on reasonable request.

## Abbreviations

CGRP: Calcitonin gene-related peptide
CGRPmAb: Anti-calcitonin gene-related peptide monoclonal antibodies
EM: Episodic migraine
CM: Chronic migraine
RR: Responder rate
RW: Real-world
ICHD-3: International Classification of Headache Disorders 3rd edition
GAD-7: Generalized Anxiety Disorder-7
PHQ-9: Patient Health Questionnaire-9
MMD: Monthly migraine days
MHD: Monthly headache days
AMD: Monthly acute medication days
NRS: Numerical rating scale
MOH: Medication-overuse headache
CI: Confidence interval
