# Supplementary material for: Real-world evidence of fremanezumab for treating migraine in Japan: a retrospective study
Source: BMC Neurol. 2023 Nov 14;23:404. doi: 10.1186/s12883-023-03449-3 (PMC10644569; doi:10.1186/s12883-023-03449-3)
Supplement: Supplementary file 1 — Additional file 1: Supplementary Figure 1. Questionnaire. [file 12883_2023_3449_MOESM1_ESM.pdf]

・Anti-CGRP monoclonal antibody: Follow-up

Reference diagram

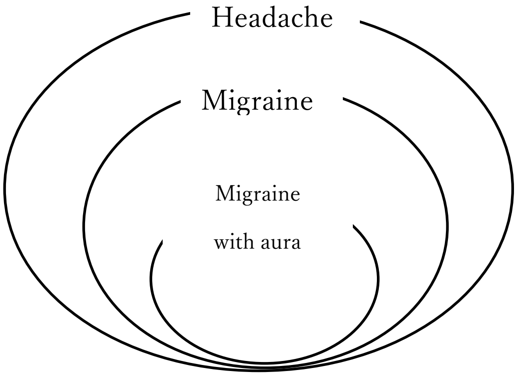

●Please tell us about your condition in the past month (28 days)

Q1. Please state the number of days with headache

( ) days

* The number of days with headache includes the number of days with migraine

Out of them, how many were days with migraine?

( ) days

Out of them, on how many days did you experience migraine with aura (visual symptoms such as scintillating scotoma)?

Scintillating scotoma: Lasts 5-60 minutes in general

( ) days

Q2. On how many days did you use acute medication?

( ) days

Q3. After taking acute medication, on average, for about how many hours did headache persist?

( ) hours

Q4. With 10 points as the maximum pain you can imagine, and 0 point as no pain, how would you rate the strength of your headaches, on average?

( ) points

Q5. Please tell us about the associated symptoms of your headache. If there were no associated symptoms, please circle "No". If you experienced any symptoms, please circle depending on the degree and severity.

Light felt dazzling	None / Mild / Moderate / Severe
Sounds felt noisy	None / Mild / Moderate / Severe
Nausea or vomiting	None / Mild / Moderate / Severe

Q6. Where did you have the injection?

Left / Right   Thigh / Stomach / Upper arm / Other (   )

Was there any reaction at the injection site?

No / Yes

If yes, please circle the most appropriate response:

Pain / Redness / Swelling / Numbness / Others (   )

In addition, how severe was the reaction?

Mild / Moderate / Severe

Q7. Are you aware of any side effects other than the reaction at the site of injection? (Please specify)

____

Q8. Did the effect of acute medication change after treatment?

Significantly improved / Improved / No changes / Worsened

Q9. Please rate your current level of satisfaction for the CGRP-related antibody drug.

Very satisfied / Somewhat satisfied / Unsatisfied

Supplementary Figure 1. Questionnaire

(a) English translation

・CGRP 関連抗体薬＿フォローアップ

●直近の 1 か月（28 日）の状況について教えてください

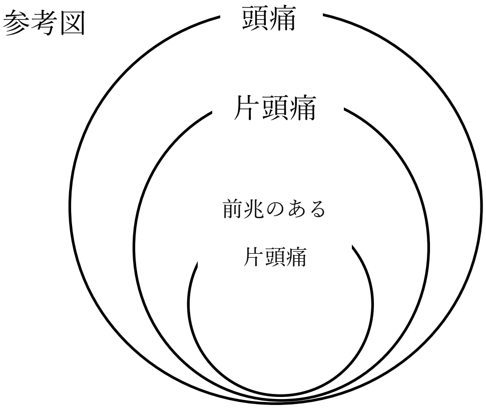

Q1. 頭痛日数を記載してください

（　　　）日

＊頭痛日数には片頭痛の日数も含みます

そのうち、片頭痛日数は何日でしたか？

（　　　）日

そのうち、前兆（閃輝暗点などの視覚症状）を伴う片頭痛は何日ありましたか？

閃輝暗点：一般的には 5～60 分続きます

（　　　）日

Q2. 急性期治療薬（頓服の鎮痛薬）は何日使用しましたか？

（　　　）日

Q3. 頭痛薬を内服した状態で、頭痛は平均で約何時間持続しますか？

（　　　）時間

Q4. 頭痛の強さについて、想像できる最大の痛みを 10、痛みがない状態を 0 とすると、平均で何点でしたか？

（　　　）点

Q5. 頭痛の随伴症状についてお伺いします。症状がない場合は、「ない」を、症状がある場合は程度に応じて、重症度に応じて○をつけてください。

光をまぶしく感じる	ない	軽度	中等度	重度
音をうるさく感じる	ない	軽度	中等度	重度
吐き気や嘔吐	ない	軽度	中等度	重度

Q6. 注射をした場所はどこですか？
左 / 右 の 太もも / おなか / 二の腕 / その他（ ）

注射をした場所に、何か反応はありましたか？

なし / あり

ありの場合： 痛み / 赤み / 腫れ / しびれ / その他（ ）
また、反応の程度はどれくらいですか？
軽度 / 中等度 / 重度

Q7. 注射部位反応以外の副作用についての自覚はありますか？（自由記載）

____

Q8. 急性期治療薬（痛いときに内服する頓服の鎮痛薬）の効果は治療後変わりましたか？

著明に改善した / 改善した / 変わりなし / 悪化した

Q9. 現時点での、CGRP 関連抗体薬に対しての満足度を評価してください

大変満足である / どちらかというと満足である / 満足ではない

Supplementary Figure 1. Questionnaire
(b) Japanese verison (original)
