# Supplementary material for: Real-world evidence of fremanezumab for treating migraine in Japan: a retrospective study
Source: BMC Neurol. 2023 Nov 14;23:404. doi: 10.1186/s12883-023-03449-3 (PMC10644569; doi:10.1186/s12883-023-03449-3)
Supplement: Supplementary file 2 — Additional file 2: Supplementary Figure 2. Responder rates at 1M for monthly and quarterly patients. [file 12883_2023_3449_MOESM2_ESM.pdf]

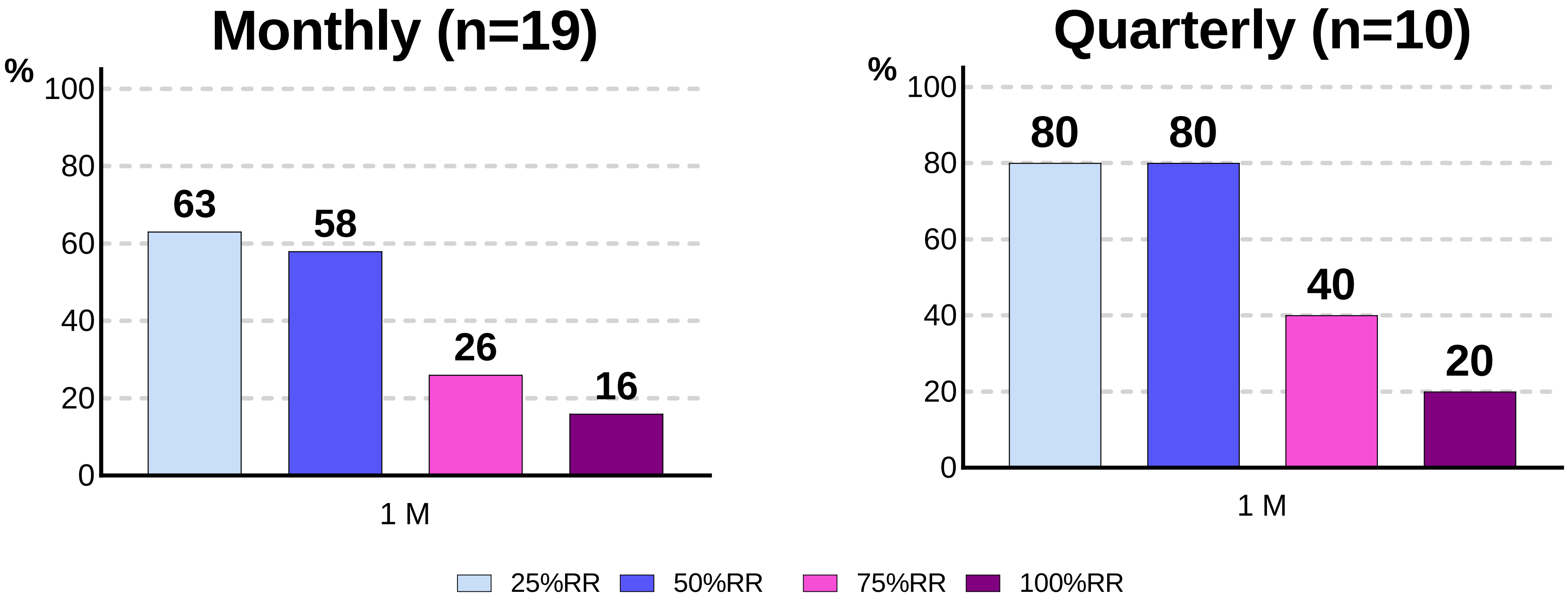

Supplementary Figure 2. Responder rates at 1M for monthly and quarterly patients
(a) Monthly patients
Patients received monthly dose of fremanezumab (225 mg) four times
(b) Quarterly patients
Patients received fremanezumab (225 mg) at first doses and quarterly doses of fremanezumab (675 mg) at second doses.
